# Mortality and Hospitalizations in Mexican Patients with Inflammatory Bowel Disease: Results from a Nationwide Health Registry

**DOI:** 10.1155/2020/8825330

**Published:** 2020-07-31

**Authors:** Andrea Sarmiento-Aguilar, María J. Ríos-Blancas, Jesús K. Yamamoto-Furusho

**Affiliations:** ^1^Inflammatory Bowel Disease Clinic, Department of Gastroenterology, National Institute of Medical Sciences and Nutrition Salvador Zubirán, Vasco de Quiroga 15, Colonia Sección XVI, Tlalpan 14080, Mexico, Mexico; ^2^Health Systems Research Center, National Institute of Public Health, Avenida Universidad 655, Santa María Ahuacatitlán 62100, Cuernavaca, Morelos, Mexico

## Abstract

**Background:**

Incidence of inflammatory bowel disease (IBD), which includes ulcerative colitis (UC) and Crohn's disease (CD), is increasing worldwide; nevertheless, it is still unknown if this is the case in Mexico. Thus, the aim of this study was to analyze the distribution and trends of hospital discharges (HD) (for the period between 2004 and 2015) and deaths (for the period between 2004 and 2013) reported for UC and CD in Mexico.

**Methods:**

Quantitative cross-sectional study was performed. Secondary data sources analysis was performed through Dynamic Cubes of the General Direction of Health Information; variables were categorized by diagnosis, age, sex, and state. The Mann–Whitney *U* test was used to analyze the differences between the first and last years that were studied. Statistical analysis was performed in SPSS v.24.

**Results:**

The number of HD increased by 98.9% between 2004 and 2015 (IBD: *p*=0.033, CD: *p*=0.009, UC: *p*=0.051); it was more frequent, for both sexes and diagnoses, between 15 and 44 years, with a second peak for men with UC (between 45 and 64 years). Deaths increased by 96.2% from 2004 to 2011 (IBD: *p*=0.056, CD: *p*=0.064, UC: *p*=0.04). UC is three times more frequent than CD. Mexico City has the highest number of HD (4,179; 22.7%) while the state of Veracruz has the highest number of deaths (273; 38.2%).

**Conclusions:**

HD for IBD in Mexico is increasing significantly; the number of deaths increased until 2011, but from then on, they are apparently decreasing. IBD affects Mexican people without any gender predominance, often affecting patients between 15 and 44 years of age. UC is three times more frequent than CD.

## 1. Introduction

Inflammatory bowel disease (IBD) is a chronic inflammatory condition of the intestine that includes Crohn's disease (CD) and ulcerative colitis (UC) which etiology is still unknown. However, it has been postulated that it is a multifactorial disease with genetics [1], environmental [2], and immunology factors [3, 4]. IBD is characterized by alternating periods of remission and relapses that can severely affect the patients' quality of life [5].

According to the study of Global Burden of Disease 2016 in Mexico, the total of disability adjusted life years (DALYs) due to IBD (16,289 (100%)), 11,903 (73.1%) are due to years of life lost (YLLs) for premature mortality and 4,386 (26.9%) are due to years lived with disease (YLDs). IBD represents 0.037% of the total YLDs in Mexico for any cause, which is similar to the YLDs caused by maternal abortion (0.036%), uterine cancer (0.03%), ovarian cancer (0.034%), and colon and rectal cancer (0.047%). The highest rates of YLDs from IBD in the world come from the United Kingdom (92.0 YLDs per 100,000), United States (US) (73.5 YLDs per 100,000), and Croatia (42.7 YLDs per 100,000). Mexico has 3.41 YLDs per 100,000. In terms of death rates, the highest comes from Germany (2.64 deaths per 100,000), Belgium (2.41 deaths per 100,000), and the United Kingdom (2.37 deaths per 100,000), while Mexico has a death rate of 0.34 deaths per 100,000 [6].

IBD is considered an expanding global health problem, as its incidence and prevalence have increased constantly through the last 20 years in adults [7] and children [8]. Regarding the adult population, it is estimated that around 1 million US residents and 2.5 million European residents are affected by IBD, the majority of them were diagnosed at early stages of their lives [9]. Furthermore, between 1999 and 2010 in Canada, 5,214 new cases of IBD were registered in children under 16 years (9.68 per 100,000 children, one of the highest incidence rates of pediatric IBD in the world), with a fast increase in the population between 0 and 5 years of age [8]. If this increased incidence of IBD continues, considering a demographic transition, IBD prevalence is expected to increase from 660 to 790 per 100,000 inhabitants between 2015 and 2025 in the US [9]. During the last 20 years, an increase of IBD frequency has been identified in some regions previously considered of low incidences, such as Eastern Europe, Asia, Africa, and Latin America. Although the main cause of this increase is still unknown, the influence of lifestyle, occidental diet, the better knowledge about its pathophysiology and diagnosis, and improvement in diagnostic resources in developing countries may be influencing this increase in IBD registered cases [10–12]. Nevertheless, published data in this regard from developing countries are lacking. In Mexico, a study in 2009 concluded that the frequency of new UC cases had increased significantly in a 10-year period [12], and a recent study from a Nationwide Cohort study showed an increased incidence and prevalence of IBD in Mexico [13].

In developing nations like Mexico, population-based epidemiological studies providing incidence rates between age groups are scarce [14], but the national registries of hospital discharges and deaths include information about the diagnosis, sex, and age that can be used as an indirect parameter about the frequency of IBD distributed and changed throughout the country and across time, taking into account that the trends of hospitalizations reflect underlying trends in disease prevalence [15]. Therefore, the aim of this study was to analyze the distribution and trends of hospital discharges for the period between 2004 and 2015 and deaths for the period between 2004 and 2013 reported for UC and CD in Mexico.

## 2. Materials and Methods

This is a quantitative and cross-sectional study. Secondary data sources analysis is performed through Dynamic Cubes of the General Direction of Health Information (DGIS for its Spanish acronym) of Mexico, from 2004 to 2015. The search was done looking for Crohn's disease (K.50) or ulcerative colitis (K.51) according to their corresponding classification in the International Classification of Diseases (ICD)-10 as principal causes of hospital discharges and deaths, classified by sex, age, and state where data were registered.

Access to the DGIS information was obtained through the Dynamic Cubes platform, which is an electronic repository of national integrated databases. This platform contains information from different periods of time, related to the number of hospital discharges and deaths from different public institutions that offer health services in Mexico. For all institutions, information about hospital discharges was reported for the years between 2004 and 2015, and information about deaths was reported between 2004 and 2013. In both cases, information was reported from the following institutions: Mexican Health Ministry (*Secretaría de Salud* or SS), Instituto Mexicano del Seguro Social (IMSS), Instituto de Seguridad y Servicios Sociales de los Trabajadores del Estado (ISSSTE), Secretaría de la Defensa Nacional (SEDENA), Secretaría de Marina (SEMAR), and Petroleos Mexicanos (PEMEX).

The database was built using annual information of hospital discharges (2004–2015) and deaths (2004–2013) in every Mexican state, categorized by CD or UC, sex, and age. Data were added in order to obtain the total numbers on a nationwide scale. Afterwards, histograms and linear graphs were used to represent data and analyze their behavior through time. The same database was exported to SPSS v24, where an evaluation of data distribution was made using the Shapiro–Wilk test (*p* < 0.05), concluding in a nonnormal distribution. Thus, descriptive statistics were run using median and interquartile ranges to describe the total national and subnational numbers, and the Mann–Whitney *U* test was used to assess the differences between the years.

The total number of hospital discharges (2004–2015) and deaths (2004–2013) for each state was ordered in a descending manner through quartiles. From this list, data were categorized in states with very high, high, low, or very low number of hospital discharges and deaths. To represent the geographic distribution of data, reference maps were obtained, assigning one different color to each category.

This study was performed in accordance with the principles expressed in the Helsinki Declaration. This study was approved by the Ethical and Medical Committee from the National Institute of Medical Sciences and Nutrition Salvador Zubirán.

## 3. Results

**Figure 1:**
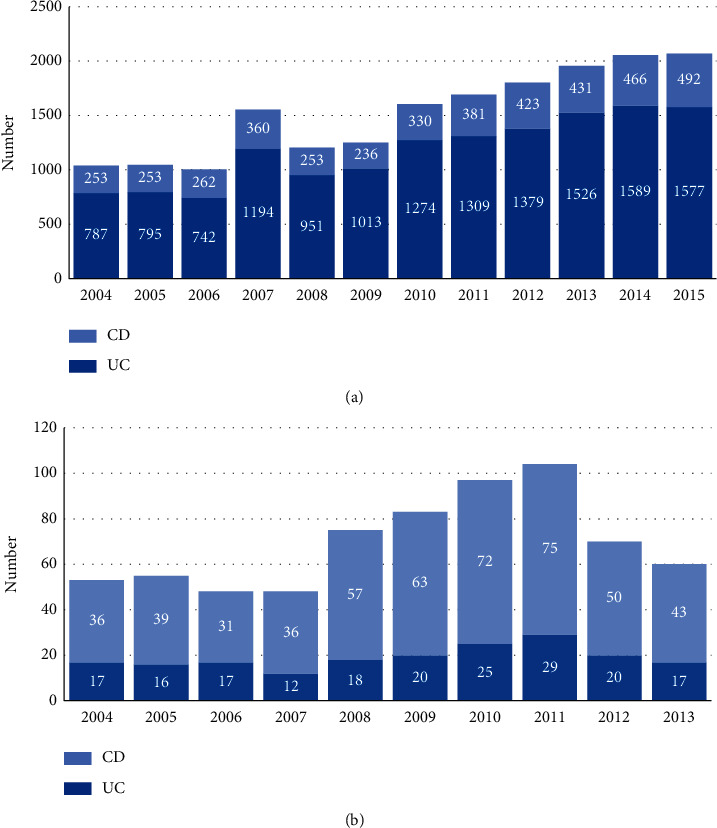
Number of hospital discharges (a) and deaths (b) reported for ulcerative colitis and Crohn's disease.

The number of HD and deaths reported for UC and CD in each one of the years studied is shown in Figure 1. The number of hospital discharges reported for UC was 3 times greater than those reported for CD throughout the same period. In 2015, UC hospital discharges represented 76.2% of the total number reported for IBD. The greatest increase in the number of hospital discharges was found for CD and UC from 2009, 108.4%, and 55.6%, respectively, which represented almost double of the increase in CD compared to UC. Considering the number of hospital discharges for both CD and UC, this increase was of 65.7% from 2009.

The number of reported deaths for CD and UC increased until 2011 but then started to decrease. The number of deaths reported for IBD in 2011 was almost two times greater than those reported in 2004. This increase was 2.08 times for UC and 1.7 times for CD. In the case of CD, the greatest increase in the number of deaths was found between 2006 and 2011 (151.7%) and its decrease afterwards was of 42.6%. On the other hand, UC had the greatest increase between 2007 and 2011 (121.4%) and a decrease afterwards of 41.4%.

**Figure 2:**
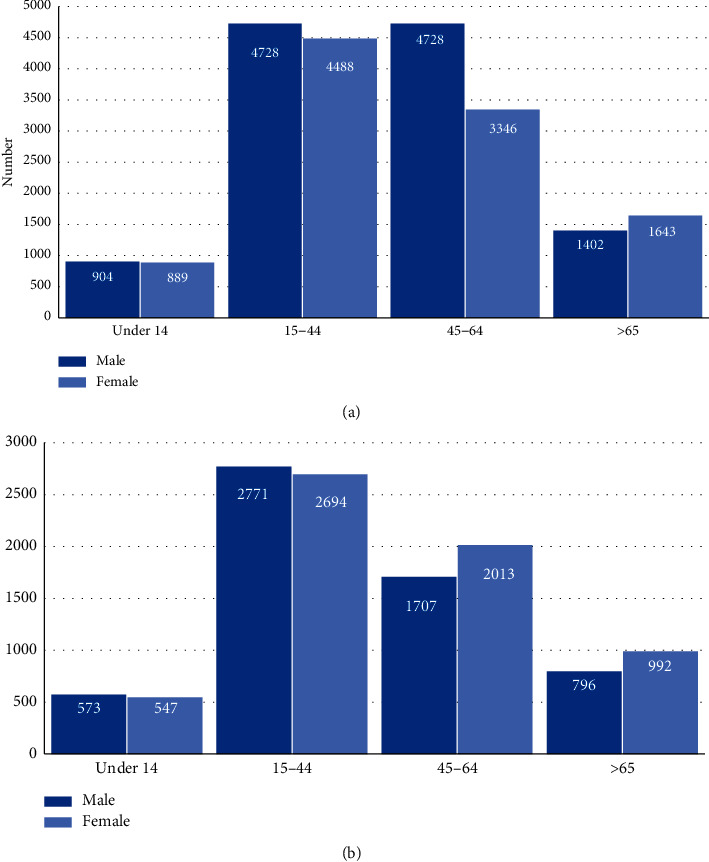
Number of hospital discharges classified by group of age and sex for ulcerative colitis (a) and Crohn's disease (b).

Figure 2 shows the number of hospital discharges categorized by age group and sex for UC (A) and CD (B). The frequency of both diagnoses is similar between men and women. The first peak of frequency in hospital discharges is observed for UC between 15 and 44 years old for both sexes (4,728 men and 4,448 women); a second peak is observed in the group from 45 to 64 years only in men with UC, which contrasts with women with UC of the same age that in this case are hospitalized less frequently (3,346 discharges). It is also observed that people between 15 and 44 years are hospitalized 5 times more frequently than people below this age and that men with UC are hospitalized 2.9 times more frequently than elderly people. On the other hand, CD had a peak of hospital discharges in both genders between 15 and 44 years (2, 771 men and 2,694 women), almost 5 times more than younger people and around 3 times more than the elderly group.

Table 1 illustrates hospital discharges or deaths from each state, categorized by the diagnosis of UC or CD and sex. All states with greater number of hospital discharges were Mexico City 4,179 (22.7%), Jalisco 1,822 (9.9%), Nuevo Leon 1,204 (6.5%), Coahuila 978 (5.3%), and Chihuahua 892 (4.8%). On the other hand, the states with the greatest number of deaths reported for IBD are Veracruz 273 (38.2%), Chihuahua 48 (6.7%), Chiapas 36 (5%), Yucatán 30 (4.2%), and San Luis Potosí 24 (3.4%).

**Figure 3:**
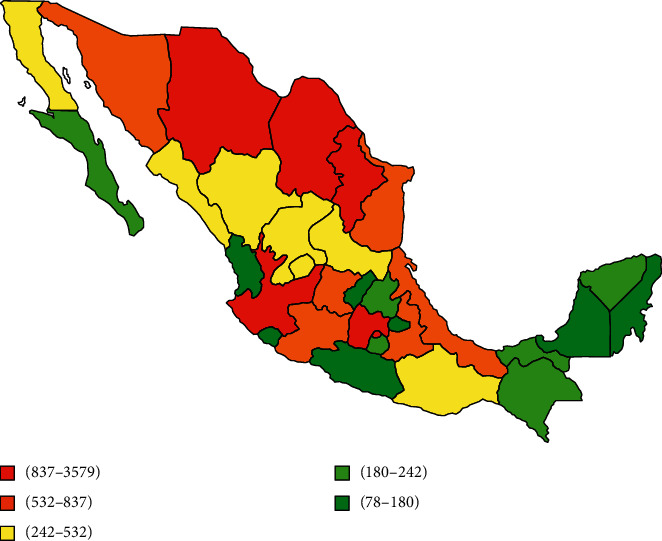
Geographic distribution of the number of hospital discharges reported in Mexico for ulcerative colitis and Crohn`s disease from 2004 to 2015.

**Figure 4:**
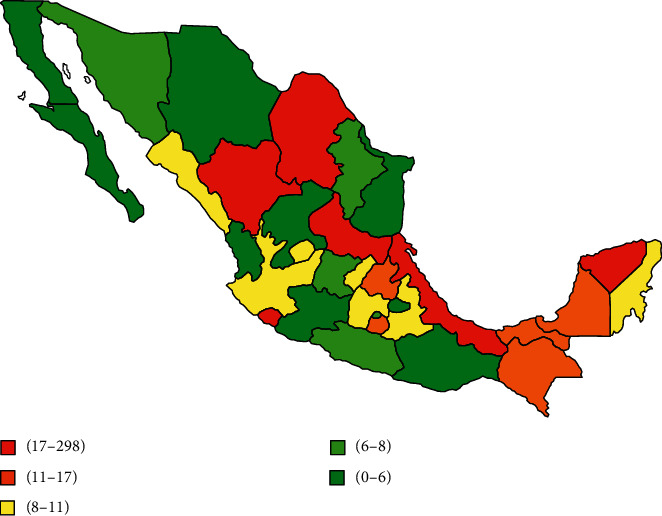
Geographic distribution of the number of hospital deaths reported in Mexico for ulcerative colitis and Crohn`s disease from 2004 to 2013.

The geographic representation of the number of hospital discharges for IBD from 2004–2015 is shown in Figure 3 and deaths from 2004–2013 are in Figure 4. The states with a greater number of hospital discharges are concentrated in the north of the country. However, this pattern is not so clear regarding reported deaths, because there are some states with a very high frequency of deaths located in the north and south of the country.

In Figure 1, it is shown that the number of hospital discharges increased by 98.9% from 2004 to 2015; in other words, it almost doubled (IBD: *p*=0.033, CD: *p*=0.009, UC: *p*=0.051).  Table 2 shows a statistically significant difference in the median of hospital discharges at 2004 compared to 2015 for CD (*p*=0.009) and IBD (*p*=0.033), while the increase in hospital discharges for UC compared with the same year showed a tendency to be significant (*p*=0.051). Concerning deaths reported for IBD, a significant increase was shown for UC (2004 vs. 2011) (*p*=0.04), whereas deaths for CD (*p*=0.064) and IBD (*p*=0.056) did not increase significantly.

Additionally, a decrease in the number of hospital discharges was reported for IBD from 2011 until 2013. This difference was only statistically significant for UC (*p*=0.05). These differences regarding CD (*p*=0.251) and IBD (*p*=0.126) were not statistically significant.

## 4. Discussion

To our best knowledge, this is the first study that showed a significant increase in the number of hospital discharges for IBD in Mexico from 2004 to 2015, while the number of deaths presented a peak in 2011 and then started to decrease. It is important to mention that IBD affects Mexican people without sex predominance, frequently between 15 and 44 years old. States with a greater number of hospital discharges were concentrated in the north and center of the country, while states with the greatest number of deaths were located in the north and south of the country.

In this study, from 2004 to 2015, although UC was three times more frequent than CD, hospital discharges for CD increased 108.4% from 2009 while for UC, there was an increase of 55.6% in the same period of time. This behavior was similar to those reported in the US, where until 2004, hospital discharges for CD increased more than 2-fold during a 35-year period, while for UC it increased only slightly [15]. The main cause of the increase in IBD registered cases is still unknown, but the influence of lifestyle, occidental diet, and better awareness of IBD in developing countries might be influencing the increase of IBD registered cases [10–12].

We found no sex predominance regarding IBD diagnosis, which is according to previous studies around the world [5, 16–18].

Hospital discharges were located in the north and center of the country, which is consistent with a previous study that reported about the severity and incidence of IBD that has been associated with geographic distribution [5]. For instance, countries located at the north of the Earth like the US, Canada, and East Europe have shown a high and stable incidence of IBD compared to those countries close to the equator that have reported low incidence such as countries in Latin America and Southern Europe [5]. Moreover, in the United States, it has also been studied that hospital discharges have increased significantly from 1994 to 2016, not only in the white race but also in black and Asian populations [19]. The reason of this fact is still unknown; nevertheless, ultraviolet ray exposure does not explain it completely [20].

Hospital death registries reported for UC in Mexico increased significantly from 2004 to 2011, whereas deaths for CD did not. The GBD study has already estimated the number of deaths caused by IBD until 2016, but if we compare the 411.59 (280.32-460-97) deaths estimated by the GBD for 2013 [6] and the 60 deaths reported in this study for the same year.

One important limitation of this study is that hospital discharges and deaths due to IBD without considering other hospital discharges or deaths in which IBD in any of its variants contributed to either of both possible outcomes without being the final main cause. Besides, this study has not considered the cases that occurred in private hospitals or outpatient clinics from public or private health services, so the number reported in this study may be underestimating the real picture.

The data considers the whole country and the majority of institutions that give health services. This represents a unique opportunity to analyze the temporary and geographic trends of IBD in Mexico. Additionally, registries from hospital discharges attributed to IBD as the principal cause could be an approximate indicator of their national frequency and can, therefore, be used as parameters for morbidity [15].

There are some potential reasons that could explain this increase in the frequency of hospital discharges and deaths attributed to CD and UC in specific regions in Mexico such as lack of IBD clinics in most of the hospitals located at countryside explained by low rate of medical treatment optimization that produces severe disease activity and consequently higher hospitalizations and surgeries related to IBD. It is important to mention that most of the hospitals belong to social security and government systems where they have access to all kinds of medical treatment including biological therapy. However, most of the specialists were afraid of using biological therapy in IBD patients.

## 5. Conclusion

This is the first Mexican study that analyzes the nationwide frequency of hospital discharges and deaths attributed to IBD in recent years. Hospital discharges increased significantly between 2004 and 2015 as well. The number of deaths had a frequency peak in 2011 and then started to decrease over time. The states with a greater number of hospital discharges were located in the north and center part of the country, while the states with a greater number of deaths were distributed both in the north and in the south of Mexico.

## Figures and Tables

**Table 1:** The number of hospital discharges and deaths reported for inflammatory bowel disease (IBD), ulcerative colitis (UC), and Crohn's disease (CD) in Mexico.

	Hospital discharges (2004-2015)										Deaths (2004-2013)									
	Women				Men				Total		Women				Men				Total	
	UC		CD		UC		CD		IBD		UC		CD		UC		CD		IBD	
	*N*	%	*n*	%	*n*	%	*n*	%	*n*	%	*n*	%	*n*	%	*n*	%	*n*	%	*n*	%
Mexico	7,496	41	2,167	11.9	6,640	36.3	1,973	10.8	18,276	100	118	16.9	244	34.9	88	12.6	250	35.7	700	100
Aguascalientes	145	1.9	24	1.1	134	2.0	22	1.1	325	1.8	4	3.4	1	0.4	3	3.4	3	1.2	11	1.6
Baja California	253	3.4	44	2.0	181	2.7	54	2.7	532	2.9	1	0.8	1	0.4	2	2.3	2	0.8	6	0.9
Baja California Sur	88	1.2	12	0.6	82	1.2	12	0.6	194	1.1	1	0.8	1	0.4	1	1.1	0	0	3	0.4
Campeche	28	0.4	18	0.8	22	0.3	10	0.5	78	0.4	5	4.2	3	1.2	3	3.4	3	1.2	14	2.0
Chiapas	92	1.2	46	2.1	66	1.0	38	1.9	242	1.3	2	1.7	7	2.9	3	3.4	5	2	17	2.4
Chihuahua	448	6.0	58	2.7	334	5.0	52	2.6	892	4.9	0	0.0	0	0.0	0	0.0	1	0.4	1	0.1
Mexico City	1,460	19.5	489	22.6	1,178	17.7	452	22.9	3,579	19.6	0	0.0	3	1.2	3	3.4	2	0.8	8	1.1
Coahuila	465	6.2	103	4.8	393	5.9	58	2.9	1,019	5.6	13	11.0	11	4.5	9	10.2	7	2.8	40	5.7
Colima	31	0.4	15	0.7	69	1.0	14	0.7	129	0.7	3	2.5	29	11.9	3	3.4	22	8.8	57	8.1
Durango	102	1.4	49	2.3	171	2.6	32	1.6	354	1.9	1	0.8	9	3.7	1	1.1	9	3.6	20	2.9
Mexico	375	5.0	88	4.1	377	5.7	71	3.6	911	5.0	0	0.0	2	0.8	1	1.1	8	3.2	11	1.6
Guanajuato	335	4.5	83	3.8	321	4.8	98	5.0	837	4.6	0	0.0	2	0.8	1	1.1	4	1.6	7	1.0
Guerrero	44	0.6	20	0.9	78	1.2	23	1.2	165	0.9	3	2.5	2	0.8	1	1.1	2	0.8	8	1.1
Hidalgo	95	1.3	23	1.1	86	1.3	27	1.4	231	1.3	1	0.8	7	2.9	1	1.1	8	3.2	17	2.4
Jalisco	865	11.5	181	8.4	676	10.2	135	6.8	1,857	10.2	0	0.0	2	0.8	3	3.4	4	1.6	9	1.3
Michoacán	236	3.1	64	3.0	242	3.6	60	3.0	602	3.3	1	0.8	0	0.0	3	3.4	2	0.8	6	0.9
Morelos	86	1.1	23	1.1	82	1.2	32	1.6	223	1.2	7	5.9	1	0.4	3	3.4	2	0.8	13	1.9
Nayarit	45	0.6	18	0.8	52	0.8	14	0.7	129	0.7	0	0.0	0	0.0	1	1.1	1	0.4	2	0.3
Nuevo León	425	5.7	165	7.6	458	6.9	161	8.2	1,209	6.6	5	4.2	1	0.4	1	1.1	1	0.4	8	1.1
Oaxaca	109	1.5	31	1.4	122	1.8	29	1.5	291	1.6	1	0.8	1	0.4	0	0.0	0	0	2	0.3
Puebla	247	3.3	49	2.3	240	3.6	64	3.2	600	3.3	5	4.2	4	1.6	2	2.3	0	0	11	1.6
Querétaro	63	0.8	17	0.8	76	1.1	24	1.2	180	1.0	5	4.2	3	1.2	1	1.1	0	0	9	1.3
Quintana Roo	46	0.6	23	1.1	39	0.6	26	1.3	134	0.7	3	2.5	1	0.4	5	5.7	2	0.8	11	1.6
Sinaloa	110	1.5	51	2.4	118	1.8	58	2.9	337	1.8	4	3.4	3	1.2	1	1.1	1	0.4	9	1.3
San Luis Potosí	153	2.0	42	1.9	128	1.9	29	1.5	352	1.9	8	6.8	4	1.6	10	11.4	6	2.4	28	4.0
Sonora	292	3.9	94	4.3	203	3.1	108	5.5	697	3.8	4	3.4	0	0.0	2	2.3	1	0.4	7	1.0
Tabasco	98	1.3	26	1.2	85	1.3	29	1.5	238	1.3	0	0.0	8	3.3	2	2.3	6	2.4	16	2.3
Tamaulipas	279	3.7	56	2.6	235	3.5	50	2.5	620	3.4	0	0.0	1	0.4	2	2.3	3	1.2	6	0.9
Tlaxcala	45	0.6	12	0.6	39	0.6	12	0.6	108	0.6	1	0.8	0	0.0	0	0.0	4	1.6	5	0.7
Veracruz	255	3.4	124	5.7	234	3.5	95	4.8	708	3.9	20	16.9	137	56.1	10	11.4	131	52.4	298	42.6
Yucatán	57	0.8	47	2.2	51	0.8	47	2.4	202	1.1	20	16.9	0	0.0	10	11.4	10	4	40	5.7
Zacatecas	124	1.7	72	3.3	68	1.0	37	1.9	301	1.6	0	0.0	0	0.0	0	0.0	0	0	0	0.0

**Table 2:** Change in the number of hospital discharges and deaths for inflammatory bowel disease (IBD) through time.

Hospital discharges						
	Median (IR)			Number		
	2004	2015	*p*	2004	2015	% of change 2004–2015
UC	18.5 (33.75–9.75)	30.5 (69.25–13.5)	0.051	787	1,577	100.3%
CD	4.5 (10.25–2.25)	9 (18–5.25)	0.009	253	492	95%
IBD	24.5 (47–13.25)	44 (87–17.75)	0.033	1040	2,069	99%

Deaths						
	2004	2011	*p*	2004	2011	% of change 2011–2013
UC	0 (1-0)	1 (1-0)	0.040	17	29	70.5%
CD	0 (1-0)	1 (1-0)	0.064	36	75	108.3%
IBD	0.5 (1-0)	1 (3-0)	0.056	53	104	96.2%

	2011	2013	*p*	2011	2013	% of change 2011–2013
UC	1 (1-0)	0 (1-0)	0.050	29	17	41.4%
CD	1 (1-0)	0 (1-0)	0.251	75	43	42.7%
IBD	1 (3-0)	0.5 (2-0)	0.126	104	60	42.3%

IR: interquartile range, UC: ulcerative colitis, CD: Crohn's disease.

## Data Availability

The data used to support the findings of this study are included within the article.

## References

[1] De Lange K. M., Barrett J. C. (2015). Understanding inflammatory bowel disease via immunogenetics. *Journal of Autoimmunity*.

[2] Van der Sloot K. W. J., Amini M., Peters V., Dijkstra G., Alizadeh B. Z. (2017). Inflammatory bowel diseases. *Inflammatory Bowel Diseases*.

[3] Baumgart D. C., Carding S. R. (2007). Inflammatory bowel disease: Cause and immunobiology. *The Lancet*.

[4] Reich K. M., Fedorak R. N., Madsen K. (2014). Vitamin D improves inflammatory bowel disease outcomes: Basic science and clinical review. *World Journal of Gastroenterology*.

[5] Malik T. A (2015). Inflammatory bowel disease: Historical perspective, epidemiology, and risk factors. *Surgical Clinics of North America*.

[6] Institute for Health Metrics and Evaluation (IHME) (2016). *GBD Compare Data Visualization*.

[7] M’Koma A. E. (2013). Inflammatory bowel disease: An expanding global health problem. *Clinical Medicine Insights: Gastroenterology*.

[8] Benchimol E. I., Bernstein C. N., Bitton A. (2017). Trends in epidemiology of pediatric inflammatory bowel disease in Canada: Distributed network analysis of multiple population-based provincial health administrative databases. *American Journal of Gastroenterology*.

[9] Kaplan G. G. (2015). The global burden of IBD: from 2015 to 2025. *Nature Reviews Gastroenterology & Hepatology*.

[10] Vegh Z., Kurti Z., Lakatos P. L. (2017). Epidemiology of inflammatory bowel diseases from west to east. *Journal of Digestive Diseases*.

[11] Senhaji N., Serrano A., Badre W. (2016). Association of inflammatory cytokine gene polymorphisms with inflammatory bowel disease in a Moroccan cohort. *Genes & Immunity*.

[12] Yamamoto-Furusho J. K. (2009). Clinical epidemiology of ulcerative colitis in Mexico. *Journal of Clinical Gastroenterology*.

[13] Yamamoto-Furusho J. K., Sarmiento-Aguilar A., Toledo-Mauriño J. J. (2019). Incidence and prevalence of inflammatory bowel disease in Mexico from a nationwide cohort study in a period of 15 years (2000–2017). *Medicine (Baltimore)*.

[14] Ruel J., Ruane D., Mehandru S., Gower-Rousseau C., Colombel J.-F. (2014). IBD across the age spectrum-is it the same disease?. *Nature Reviews Gastroenterology & Hepatology*.

[15] Sonnenberg A. (2009). Hospitalization for inflammatory bowel disease in the United States between 1970 and 2004. *Journal of Clinical Gastroenterology*.

[16] Ananthakrishnan A. N. (2015). Epidemiology and risk factors for IBD. *Nature Reviews Gastroenterology & Hepatology*.

[17] Rocchi A., Benchimol E. I., Bernstein C. N. (2012). Inflammatory bowel disease: a Canadian burden of illness review. *Canadian Journal of Gastroenterology*.

[18] Silva B. C. d., Lyra A. C., Rocha R., Santana G. O. (2014). Epidemiology, demographic characteristics and prognostic predictors of ulcerative colitis. *World Journal of Gastroenterology*.

[19] Sewell J. L., Yee H. F., Inadomi J. M. (2010). Hospitalizations are increasing among minority patients with Crohnʼs disease and ulcerative colitis. *Inflammatory Bowel Diseases*.

[20] Stein A. C., Gaetano J. N., Jacobs J. (2016). Northern latitude but not season is associated with increased rates of hospitalizations related to inflammatory bowel disease: Results of a multi-year analysis of a national cohort. *PLoS One*.

